# Comparison of genome-wide single-nucleotide polymorphism linkage analyses in Caucasian and Hispanic NARAC families

**DOI:** 10.1186/1753-6561-1-s1-s97

**Published:** 2007-12-18

**Authors:** Wei V Chen, Christopher I Amos, Carol J Etzel, Sanjay Shete, Peter K Gregersen

**Affiliations:** 1 Department of Epidemiology, U.T. M.D. Anderson Cancer Center, Houston, Texas 77030, USA; 2 Robert S. Boas Center for Genomics and Human Genetics, Feinstein Institute for Medical Research, Manhasset, New York 11030, USA

## Abstract

We performed linkage analysis on families with rheumatoid arthritis, stratifying by ethnic origin. We compared results using either Kong and Cox nonparametric LOD scores or MOD score analysis using the software GeneHunter MODSCORE. We first applied SNPLINK to remove markers showing excess linkage disequilibrium from the SNPs in the Illumina IV SNP Linkage panel. In this analysis there were 659 self-reported Caucasian families and 29 self-reported Hispanic families in the NARAC collection. Chromosome 19 yielded MOD scores > 3.00 in the Hispanic group, while chromosomes 2, 6, 7, 11, and XY had MOD scores > 3.00 in the Caucasian group. We performed simulation studies to evaluate the empirical distribution of the MOD score for autosomal loci separately in Hispanics and Caucasians. Results showed genome-wide significant evidence for linkage in Caucasians for chromosomes 2q and 6p, but no significant evidence for any linkages in the Hispanics, including little evidence for linkage to chromosome 6p in this group. An examination of the difference of phenotypes in two ethnic groups suggested significantly earlier mean age of onset, higher percentage of anti-cyclic citrullinated peptide positive people, and lower percentage of affected people carrying shared epitopes in Hispanics than those in Caucasians. A larger sample size of the Hispanic group is needed to identify linkage regions.

## Background

Rheumatoid arthritis (RA) is a complex genetic disease with possible genetic heterogeneity among different ethnic groups [1]. There is a lack of information concerning genetic risk factors for RA in Hispanic populations, so we sought to characterize in the available sample both the clinical features as well as the genetic profiles that influence disease risk. We evaluated the phenotypes between the two groups to compare their ages of onset, rheumatoid factor (RF)-IgM and anti-CCP (anti-cyclic citrullinated peptide) levels and the percentage of affected individuals carrying shared epitopes.

Previously we performed a genome-wide single-nucleotide polymorphism (SNP) analysis of NARAC (North American Rheumatoid Arthritis Consortium) Caucasian families and identified two new loci at 2q33 and 11p12, in addition to confirming evidence for linkage in the HLA region (Kong and Cox LOD score of 16.14) [2].

Here we applied standard linkage analysis methods as well as the MOD score approach to this same set of Caucasian families as well as to a set of Hispanic families to compare evidence of linkage to RA in these two groups. In addition, the MOD score method provides estimates of the penetrance for putative disease-susceptibility loci, while conditioning on the disease status, thus adjusting for ascertainment of the families. However, the distribution of the MOD score test statistic is complex, and we therefore have performed extensive simulations to obtain empirical *p*-values.

Evidence of linkage on chromosomes 2 and 6 was confirmed by MOD score analysis for the Caucasian group, and weak evidence of linkage to chromosome 6 was found to be not significant in Hispanic group using empirical *p*-values. Significant differences of phenotypes between these two groups were found in age of onset, proportion of anti-CCP positive people, and percentage of affected people carrying the shared epitopes.

## Methods

An *R*^2 ^value, a measure of linkage disequilibrium (LD), of 0.05 was used as cut-off to remove markers of linkage disequilibrium (LD) using SNPLINK [3,4]. The data sets for both Caucasians and Hispanics were analyzed by SNPLINK with Merlin [3,5] and by GeneHunter using MODSCORE [6].

To assess significance in the Hispanic sample, we performed simulations of the families for all chromosomes (excluding the X chromosome) using 10,000 replicate samples with the computer program Allegro [7]. To derive a genome-wide estimate of the maximum MOD score, we selected from each replicate the maximum MOD score across all autosomal loci. Owing to the computational intensity of the simulations in the much larger Caucasian sample, we could only complete simulation of 1000 replicates. To obtain more precise estimates of the empirical MOD score distribution we derived 100,000 bootstraps from the simulated results for Caucasians to obtain a distribution of MOD scores in this group. To perform the bootstrap, we randomly selected a maximum MOD score from each chromosome from each of the 1000 replicated results, and then selected the maximum MOD score from all 22 chromosomes to obtain the maximum genomic MOD score for that replicate. This process was repeated 100,000 times to obtain empirical MOD score distribution.

Distributions of phenotypes, including age of onset, anti-CCP, RF-IgM and shared epitopes, were also compared between two groups. The significance of the differences were formally assessed using a *t*-test, binomial test, or survival modeling, since segregation analysis cannot be done because of the complex ascertainment used for selecting these families. We know who the primary proband is but not who the obligatory additional proband is. Therefore, we have chosen to apply a MOD score approach, which conditions on the disease status in the family and subsequently performs a segregation analysis to estimate parameters describing penetrance.

## Results

The results presented in Table 1 show maximal Kong and Cox (KC) LOD scores and MOD scores on each chromosome separately for Caucasians and for Hispanics. For Caucasians, maximal KC LOD scores exceeding 3.00 are found on chromosomes 2, 6, 11, and the pseudoautosomal region of XY (we did not study this region further because results from Amos et al. [2] suggest the XY region reflects a false-positive signal). MOD score analysis indicated identical positions and slightly higher scores for each of these chromosomes. Contrasting results of MOD score and KC LOD score analyses in Hispanics showed no KC LOD scores over 1.50, but 9 chromosomes yielded MOD scores higher than 1.50. Of note, chromosome 6 showed only weak evidence for linkage in Hispanics using either KC LOD score or MOD score methods. MOD score analysis suggested evidence for linkage on chromosome 19, which was not provided in the LOD score analysis, suggesting a possible false-positive result. Best fitting models from MOD score analyses corresponding to maximal MOD scores of 1.50 or greater are provided in Table 2 for Caucasians and Hispanics, even though they often lead to excess predicted prevalence of the disease.

**Table 1:** Peak MOD scores versus peak LOD scores for both Caucasians and Hispanics

	Caucasians				Hispanics			
		
	KC LOD		MOD		KC LOD		MOD	
				
Chromosome	Value	Position	Value	Position	Value	Position	Value	Position
1	1.51	216.63	**1.60**^a^	216.62	0.60	143.47	**1.87**	234.18
2	3.51	192.6	**4.10**	192.6	0.15	225.83	1.27	210.49
3	0.58	16.82	0.86	139.58	1.27	78.83	**2.03**	57.93
4	2.47	105.02	**1.77**	16.9	0.85	83.21	**2.64**	79.01
5	2.33	44.87	**2.58**	44.86	0.28	151.77	1.23	163.90
6	16.19	32.99	**16.87**	32.99	0.82	15.12	**2.09**	15.12
7	1.98	135.69	**3.22**	135.68	0.94	13.40	1.37	38.65
8	0.93	106.47	0.92	106.47	0.9	138.39	1.13	136.91
9	0.66	72.63	**1.66**	105.82	0.35	127.63	**1.73**	127.07
10	2.61	70.4	**2.85**	70.4	1.20	82.04	**2.39**	43.55
11	3.31	41.03	**3.70**	41.03	0.20	22.36	1.01	62.74
12	1.45	22.22	**1.97**	24.4	0.01	3.27	0.38	105.69
13	0.71	33.99	1.21	22.82	0.41	27.13	**1.50**	22.99
14	0.34	80.63	**2.16**	75.58	0	99.27	1.44	54.70
15	0.32	45.39	0.70	50.72	0.71	58.43	1.10	58.43
16	1.45	53.41	**1.73**	58.85	0.34	7.72	1.01	7.72
17	1	67.57	1.11	67.56	0.08	0.09	0.56	92.56
18	1.22	52.34	1.42	39.73	1.11	62.08	**1.61**	62.08
19	0.08	61.19	0.55	41.62	0.42	7.04	**3.03**	7.04
20	0.99	55.17	1.39	56.47	0.52	63.38	1.02	39.42
21	1.32	40.85	1.42	37.43	0.37	31.45	0.64	31.45
22	0.33	39.12	0.96	43.33	0.05	48.64	0.24	60.45
XY	3.99	153.44	**4.88**	153.44	-0.01	153.44	0.32	153.66

^a^MOD scores ≥ 1.50 are bolded.

**Table 2:** Best MOD score^a ^models for peak locations in both Caucasians and Hispanics

Caucasians					Hispanics				
	
Chr	*p*	f+/+	fm/+	fm/m	Chr	*p*	f+/+	fm/+	fm/m
1	0.19000	0.0300	0.0900	0.0700	1	0.26000	0.2600	0.0004	1.0000
2	0.10000	0.0450	0.1700	0.2800	3	0.00060	0.0020	0.0300	1.0000
4	0.09000	0.1000	0.0500	0.5200	4	0.00003	0.0020	0.9900	1.0000
5	0.50000	0.0500	0.0300	0.1200	6	0.01500	0.0000	0.9900	0.0000
6	0.25000	0.0000	0.3900	0.5600	9	0.01500	0.0000	0.9900	0.9800
7	0.04500	0.0500	0.1800	0.0000	10	0.00100	0.0020	0.0000	0.3500
9	0.00040	0.0250	0.1200	1.0000	13	0.00200	0.0020	0.0000	0.3500
10	0.42000	0.0000	0.0700	0.1200	18	0.00030	0.0020	0.1500	0.2800
11	0.46000	0.0000	0.5800	0.3100	19	0.00006	0.0001	0.0000	0.9700
12	0.03500	0.0350	0.1200	0.0000					
14	0.03500	0.1100	0.0080	1.0000					
16	0.04500	0.0350	0.1100	0.1900					
XY	0.06000	0.0200	0.1000	0.0700					

^a^MOD scores ≥ 1.50

**Figure 1:**
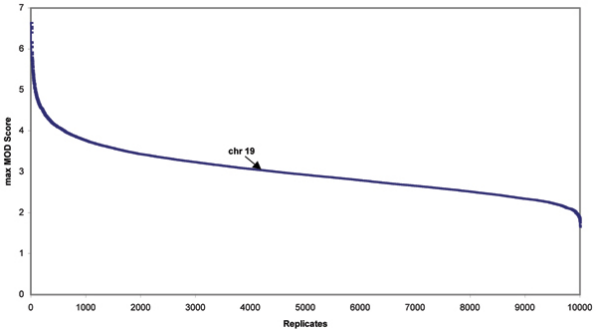
**Distribution of genome-wide maximum simulated MOD scores in each of 10,000 replicate for the Hispanics**. In the genome-wide simulated data of all 22 autosomal chromosomes, a maximum MOD score of 3.03 from the real Hispanic data on chromosome 19 (as pointed by arrow) corresponds to a *p*-value of 0.42.

To better characterize the results that we obtained from MOD score analysis suggesting linkage on chromosome 19 in Hispanics, we performed a genome-wide simulation study with 10,000 replicates. Figure 1 presents a distribution of maximum MOD scores among all 22 autosomal chromosomes of each of the 10,000 replicates from genome-wide simulation data. A max MOD score of 3.03 corresponds to a genome-wide *p*-value of 0.42, suggesting that evidence on chromosome 19 was a false-positive finding for Hispanics.

**Figure 2:**
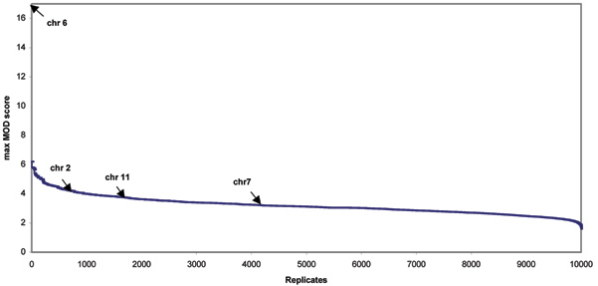
**Distribution of genome-wide simulated MOD scores in 100,000 bootstraps on 1000 replicates for the Caucasians**. Every tenth genome-wide maximum simulated MOD scores by bootstrapping 100,000 times on 1000 replicates are used in the figure due to the number of points limited for plotting. Maximum MOD scores of 16.87, 4.10, 3.70, and 3.22 from real Caucasian data on chromosomes 6, 2, 11, and 7 correspond to genome-wide *p*-values of ~0.0, 0.08, 0.17, and 0.42, respectively, as indicated by arrows.

We also performed a similar simulation study for Caucasians using only 1000 replicates because of the computational burden – more than 20 days of CPU time per chromosome would be needed and therefore a genome-wide study of 10,000 replicates is computationally prohibitive, since the sample size for the Caucasian group is more than 20 times larger than that of Hispanic group. The results are summarized in Figure 2. Max MOD scores of 16.87, 4.10, 3.70, and 3.22 on chromosomes 6, 2, 11, and 7 correspond to genome-wide *p*-values of ~0.0, 0.08, 0.17, and 0.42, respectively. The empirically derived MOD scores corresponding to *p*-values of 0.05 in Hispanics and Caucasians deviated somewhat. For Caucasians, the empirical MOD score for 5% significance is 4.39 while for Hispanics it is 4.08.

Comparison of the distributions of phenotypes including age of onset, anti-CCP, RF-IgM, and shared epitopes between two groups were included in Table 3. The Hispanic group was found to have earlier age of onset (mean 34.48 vs. 39.35), higher anti-CCP values (mean 120.20 vs. 107.51), higher RF-IgM values (mean 319.10 vs. 255.06), and a higher percentage of anti-CCP positive people (anti-CCP ≥ 20, 91.53% vs. 76.45%, results not shown) yet a smaller percentage of affected people carried the shared epitopes (71.88% vs. 84.55%). The differences in age of onset were compared using survival analysis using robust variance correction for controlling familial correlation of the ages. This analysis suggested significantly different hazard risks between Hispanics and Caucasians (*p*-value = 0.02 in Cox regression analysis after correction). The means of anti-CCP and RF-IgM of two groups were compared using *t*-test and was found not significant (*p*-value = 0.18 and *p*-value = 0.20 for anti-CCP and RF-IgM, respectively). However, the difference in proportion of anti-CCP-positive individuals in Hispanics and Caucasian is highly significant using the binomial test (two-sided exact *p*-value = 0.0032). The difference of percentage of shared epitopes in affected people is also significant (two-sided exact *p*-value = 0.01 using binomial test).

**Table 3:** Comparison of distribution of phenotypes in Caucasians and Hispanics

	Caucasians		Hispanics	
		
Phenotypes	mean ± SD	median	mean ± SD	median
Age at onset (yr)	39.35 ± 13.38	39	34.48 ± 12.42	35
Anti-CCP^a^	107.51 ± 79.44	111	120.20 ± 72.66	139
RF-IgM^a^	255.06 ± 568.52	77	319.10 ± 386.38	127.5
				
Shared epitopes	84.55%		71.88%	

^a^See Criswell et al. [9] for details on sample quantitation

The mean number of affected siblings in the families is about the same in both groups (2.10 and 2.13 for Hispanics and Caucasians, respectively). The proportion with parents available might affect MOD score calculations, but is actually higher in Hispanics (65.52%) than in Caucasians (41.09%). Allele frequencies for the SNPs giving high Kong and Cox LOD scores or MOD scores on chromosomes 6 and 19 did not reveal significant difference between the two ethnic groups (data not shown here). There were no detectable genotyping errors in these ethnic groups.

## Discussion

The results may reflect underlying genetic variations between Caucasian and Hispanic groups useful for diagnosis and treatment of disease. The sample size of Hispanics available for study strongly limits our ability to generalize our findings. Using the Caucasian sample as standard, the expected LOD score in Hispanics from 29 families is 0.71, so the evidence for linkage to chromosome 6 is comparable to its expectation. However, we note that chromosomes other than 6 yielded higher LOD scores in the Hispanics, suggesting that non-HLA-region genes may play a stronger role in this population than in Caucasian populations. The weaker linkage to HLA and lower percentage of people carrying shared epitopes in Hispanics are interesting because the associations in that group with the shared epitopes are quite weak and tend to yield lower associations in general [8]. There also may be genetic heterogeneity in Hispanic and Caucasian groups. It is important to note that the regions that were identified by KC-LOD score and MOD score were consistent in Caucasian families but not in the Hispanic family data. This difference also could be due to the small number of families in the Hispanic group. The sample size may be important when performing MOD score analyses because MOD scores are optimized over several parameters.

## Conclusion

Based on empirical *p*-values obtained from genome-wide simulations, only chromosome 6 HLA region showed very significant linkage, and chromosome 2 showed suggestive linkage to RA in the Caucasian group. MOD score analysis did not yield evidence for any new linkages and failed to provide significant evidence of linkage to chromosome 11, which has been suggested based upon standard linkage analysis.

Further research with a larger sample size of the Hispanics group is needed to confirm our findings, which suggest phenotypic and linkage heterogeneity in this group compared to Caucasians.

## Competing interests

The author(s) declare that they have no competing interests.
